# Supplementary material for: Limited changes in locomotor recovery and unaffected white matter sparing after spinal cord contusion at different times of day
Source: PLoS One. 2021 Nov 23;16(11):e0249981. doi: 10.1371/journal.pone.0249981 (PMC8610253; doi:10.1371/journal.pone.0249981)
Supplement: S1 Methods — (DOCX) [file pone.0249981.s001.docx]

**Supplementary Methods**

*Spinal cord injury and post-surgery animal care*. Twenty-two eight-week-old C57Bl/6 wild-type female mice (17–20 g of body weight, Jackson Laboratory, Bar Harbor, ME) were anaesthetized by an i.p. injection of 400 mg/kg body weight 2,2,2-tribromoethanol. Lacri-Lube ophthalmic ointment (Allergen, Irvine, CA) was applied to prevent drying of eyes and 1 ml saline s.c. to prevent animal dehydration. Moderate contusion injuries (50 kdyn force/400–600 μm displacement) were performed at the T9 level using the IH impactor (Infinite Horizons, Lexington, KY) following a laminectomy at T9 vertebrae. Muscle layers were immediately sutured (J422H, Ethicon, Sommerville, NJ), and the skin wound was closed by 9 mm veterinary stainless steel clips (EZC- Kit, Stoelting, Wood Dale, IL) followed by application of Bacitracin antibiotic ointment (Perrigo, Allegan, MI). At all steps toe, tail or back skin pinching method was used to verify the depth of anesthesia. Animals were maintained on the heating pads during surgical procedures and the 24h-postsurgery period (37^o^C) to avoid the hypothermia. Postoperative care included s.c. 1 ml saline (immediately after surgery), and 0.1 mg/kg s.c. buprenorphine every 12 h for 2 days, and manual expression of bladders twice a day for seven to ten days or until spontaneous voiding returned. All surgical and post-operation procedures were completed according to NIH and IACUC guidelines. Veterinary MUSICC Identification System employing 12mm Sterilizable Integrated Chip Carrier (#2023), and MiniTracker I chip reader device (#1002, AVID, Norco, CA) was used to identify individual animals and maintain blinded studies. At the end of the study animals were euthanized by deep anaesthesia (400 mg/kg body weight 2,2,2-tribromoethanol) and transcranial perfusion with ice cold phosphate buffered saline (PBS, 30 ml/mouse), and 4% paraformaldehyde (PFA, pH 7.4 in PBS, 30 ml/mouse) and the spinal cord tissue was dissected. During the study, no symptoms of serious complications (such as lethargy, no signs of coat grooming, dehydration) were observed in any animal. Therefore, no humane endpoint euthanasia was needed except one mouse with an accidental bladder rupture during post-SCI bladder expression. Two other mice that were lost during the study were found dead without any prior signs of serious sickness.
