# Supplementary material for: Limited changes in locomotor recovery and unaffected white matter sparing after spinal cord contusion at different times of day
Source: PLoS One. 2021 Nov 23;16(11):e0249981. doi: 10.1371/journal.pone.0249981 (PMC8610253; doi:10.1371/journal.pone.0249981)
Supplement: S1 Table — (DOCX) [file pone.0249981.s003.docx]

**Supplementary Table S1. Contusion parameters for individual animals**

|  | **Force [kdyn]** | |  |  |  | **Displacement [µm]** | |  |  |  | **Velocity [mm/s]** | |  |  |
| --- | --- | --- | --- | --- | --- | --- | --- | --- | --- | --- | --- | --- | --- | --- |
| **SCI at** | **ZT0** | **ZT6** | **ZT12** | **ZT18** |  | **ZT0** | **ZT6** | **ZT12** | **ZT18** |  | **ZT0** | **ZT6** | **ZT12** | **ZT18** |
|  | 50 | 51 | 51 | 53 |  | 652 | 846 | 687 | 599 |  | 124 | 129 | 127 | 129 |
|  | 50 | 51 | 49 | 52 |  | 546 | 388 | 564 | 546 |  | 129 | 124 | 129 | 120 |
|  | 50 | 52 | 49 | 53 |  | 599 | 529 | 546 | 458 |  | 122 | 120 | 122 | 124 |
|  | 53 | 51 | 50 | 50 |  | 546 | 582 | 423 | 493 |  | 117 | 117 | 120 | 120 |
|  | 50 | 51 | 50 | 52 |  | 476 | 546 | 529 | 546 |  | 122 | 129 | 124 | 129 |
|  | 51 | 51 | 50 | 50 |  | 546 | 546 | 635 | 599 |  | 117 | 129 | 122 | 124 |
|  | 51 | 49 | 50 | 52 |  | 564 | 458 | 564 | 652 |  | 117 | 124 | 117 | 122 |
|  | 51 | 51 | 51 | 50 |  | 546 | 493 | 652 | 564 |  | 117 | 129 | 122 | 127 |
|  | 54 | 53 |  | 52 |  | 440 | 493 |  | 493 |  | 122 | 129 |  | 129 |
|  | 51 | 51 |  | 50 |  | 758 | 511 |  | 546 |  | 129 | 124 |  | 129 |
|  |  |  |  |  |  |  |  |  |  |  |  |  |  |  |
| **average** | **51.1** | **51.1** | **50** | **51.4** |  | **567.3** | **539.2** | **575** | **549.6** |  | **121.6** | **125.4** | **122.875** | **125.3** |
| stdev | 1.37032 | 0.994429 | 0.755929 | 1.264911 |  | 88.76442 | 120.496 | 83.13157 | 58.12859 |  | 4.718757 | 4.351245 | 3.796145 | 3.772709 |
|  |  |  |  |  |  |  |  |  |  |  |  |  |  |  |
| **one-way ANOVA** |  |  | **p=0.077217** | |  |  |  | **p=0.826689** | |  |  |  | **p=0.139885** | |

Due to an accidental data loss, contusion parameters were not available for one animal of the ZT12 group. No significant differences in any contusion parameter were observed between the groups (one way ANOVA).
