# Supplementary material for: Limited changes in locomotor recovery and unaffected white matter sparing after spinal cord contusion at different times of day
Source: PLoS One. 2021 Nov 23;16(11):e0249981. doi: 10.1371/journal.pone.0249981 (PMC8610253; doi:10.1371/journal.pone.0249981)
Supplement: S2 Table — (DOCX) [file pone.0249981.s004.docx]

**Supplementary Table S2. Nucleotide sequence of qPCR primers**

| **Gene** | **Primer sequences** |
| --- | --- |
| *Bmal1 (Arntl)* | Forward: 5’-GCAGTGCCACTGACTACCAAGA-3’  Reverse: 5’-TCCTGGACATTGCATTGCAT-3’ |
| *Clock* | Forward: 5’-GCCTCAGCAGCAACAGCAGC-3’  Reverse: 5’-ACCGCATGCCAACTGAGCGA-3’ |
| *Npas2* | Forward: 5’-TGGCCTGAGCCTCACCACGA  Reverse: 5’-GCAACAGCCTGAGCTGCCGA |
| *Nr1d1*  *(Rev-erb-α)* | Forward: 5’-ACGACCCTGGACTCCAATAA-3’  Reverse: 5’-CCATTGGAGCTGTCACTGTAGA-3’ |
| *Nr1d2*  *(Rev-erb-*β*)* | Forward: 5’-ACGGATTCCCAGGAACATGG-3’  Reverse: 5’-CCTCCAGTGTTGCACAGGTA-3’ |
| *Cry1* | Forward: 5’-CCCAGGCTTTTCAAGGAATGGAACA-3’  Reverse: 5’-TCTCATCATGGTCATCAGACAGAGG-3’ |
| *Cry2* | Forward: 5’-GCTGGAAGCAGCCGAGGAACC-3’  Reverse: 5’-GGGCTTTGCTCACGGAGCGA-3’ |
| *Per1* | Forward: 5’-TCCTCCTCCTACACTGCCTCT-3’  Reverse: 5’-TTGCTGACGACGGATCTTT-3’ |
| *Per2* | Forward: 5’-CAACACAGACGACAGCATCA-3’  Reverse: 5’-TCCTGGTCCTCCTTCAACAC-3’ |
| *Per3* | Forward: 5’-CTGCTCCAACTCAGCTTCCTTT-3’  Reverse: 5’-TTAGACAGCAAGGCTCTGGTTCT-3’ |
| *Dbp* | Forward: 5’-AATGACCTTTGAACCTGATCCCGCT-3’  Reverse: 5’-GCTCCAGTACTTCTCATCCTTCTGT-3’ |
| *Gapdh* | Forward: 5’-AGGTCGGTGTGAACGGATTTG-3’  Reverse: 5’-TGTAGACCATGTAGTTGAGGTCA-3’ |
| *B2m* | Forward: 5’-GGTCTTTCTGGTGCTTGTCTCA-3’  Reverse: 5’-GTTCGGCTTCCCATTCTCC-3’ |
