# Supplementary figures and images for: Limited changes in locomotor recovery and unaffected white matter sparing after spinal cord contusion at different times of day
Source: PLoS One. 2021 Nov 23;16(11):e0249981. doi: 10.1371/journal.pone.0249981 (PMC8610253; doi:10.1371/journal.pone.0249981)

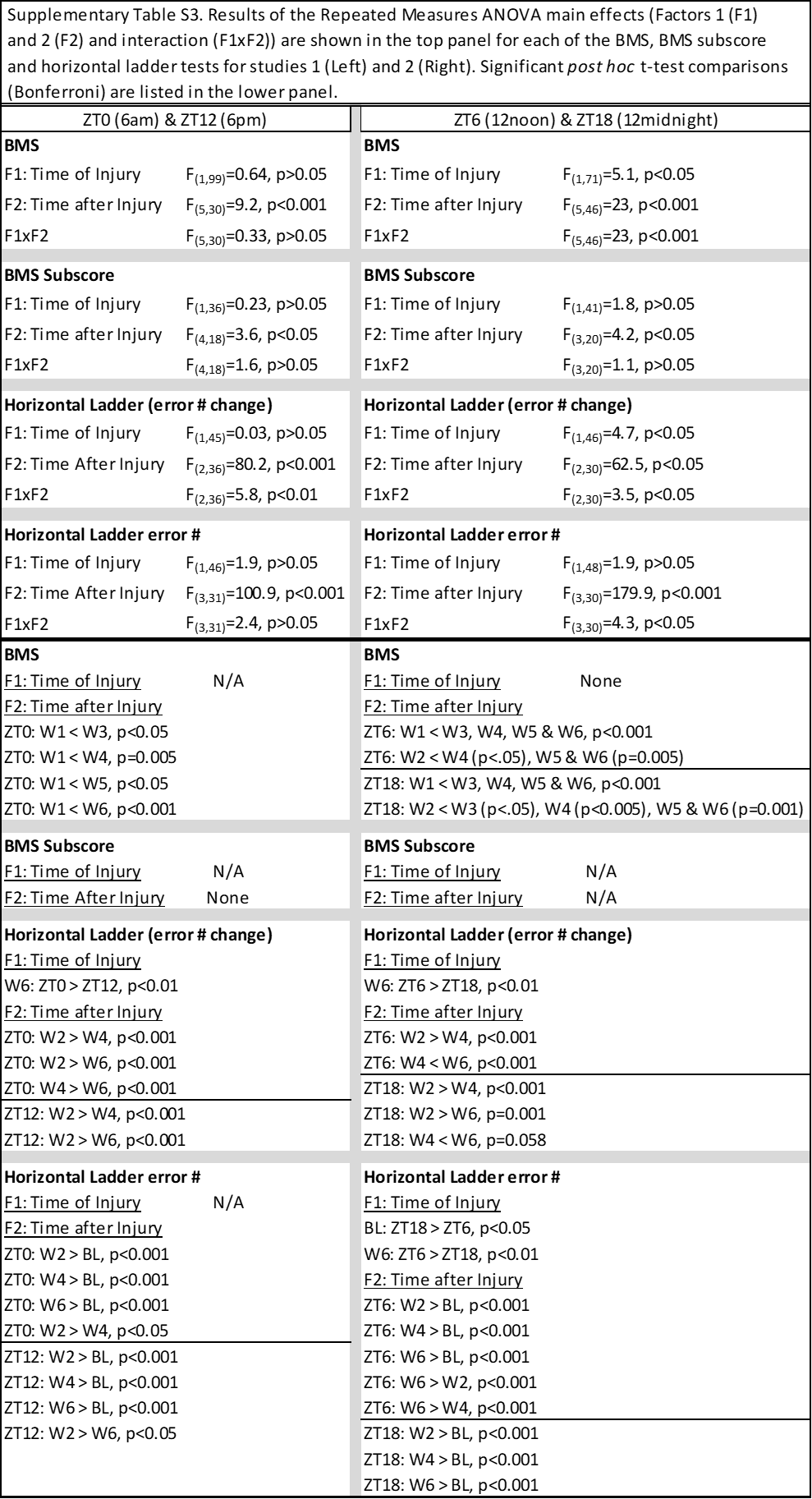

Supplement: S3 Table — (DOCX) [file pone.0249981.s005.docx]

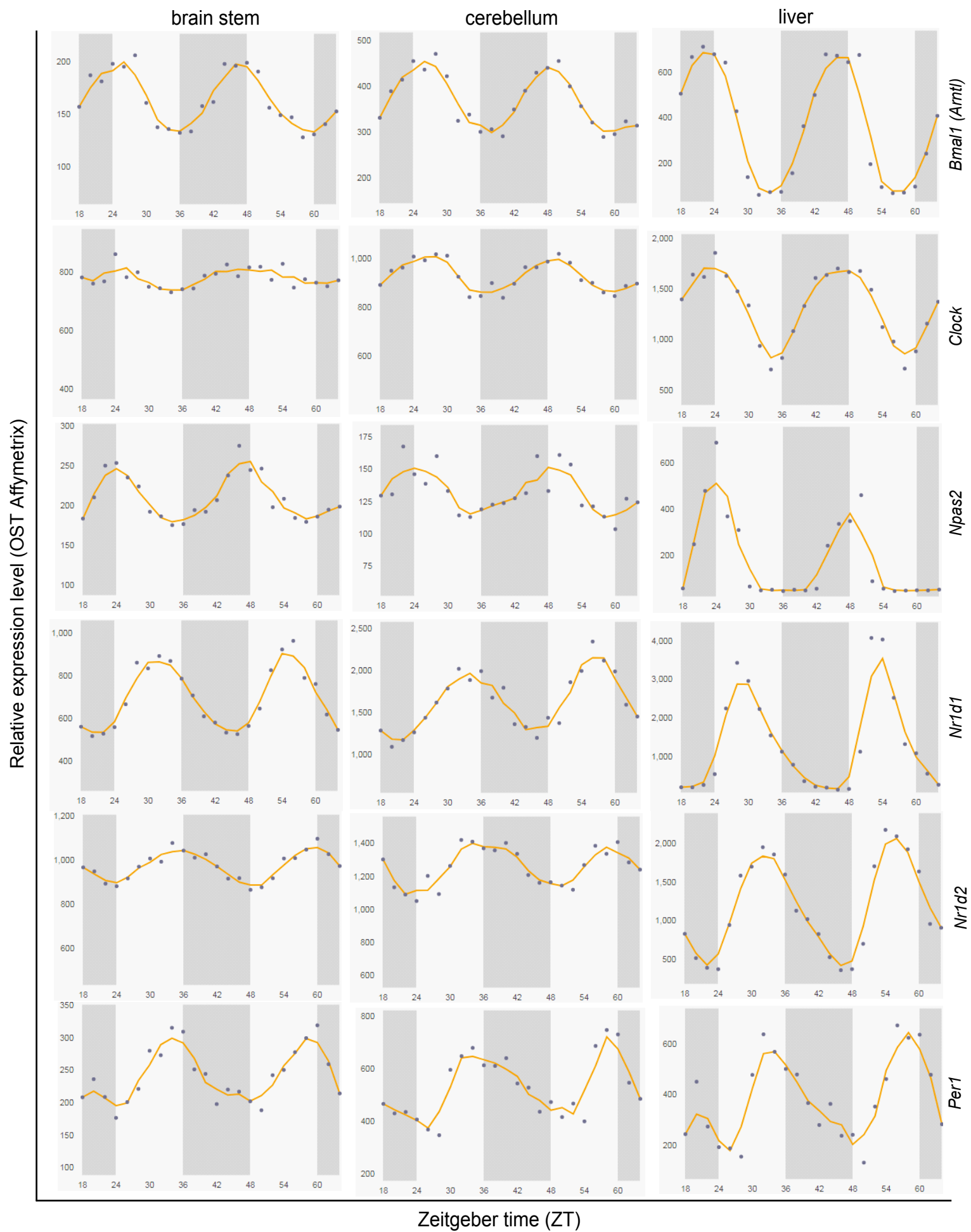

Supplement: S1 Fig — The data are from the publicly available circadian transcriptome database (http://circadb.hogeneschlab.org/mouse). All presented mRNAs show significant circadian oscillations in all three tissues (JTK p<0.05) except Cry1 (non-significant cycling in the brain stem /JTK p = 0.058/) and B2m (no cycling in any tissue /JTK p = 1/). Note that the presented default output graphs from the circadb database show one full 24 h period from ZT24 though ZT48. Therefore, ZT1 or ZT12 in Fig 1 corresponds to ZT25 or 36 in S1 and S2 Figs, respectively. (PDF) [file pone.0249981.s006.pdf]

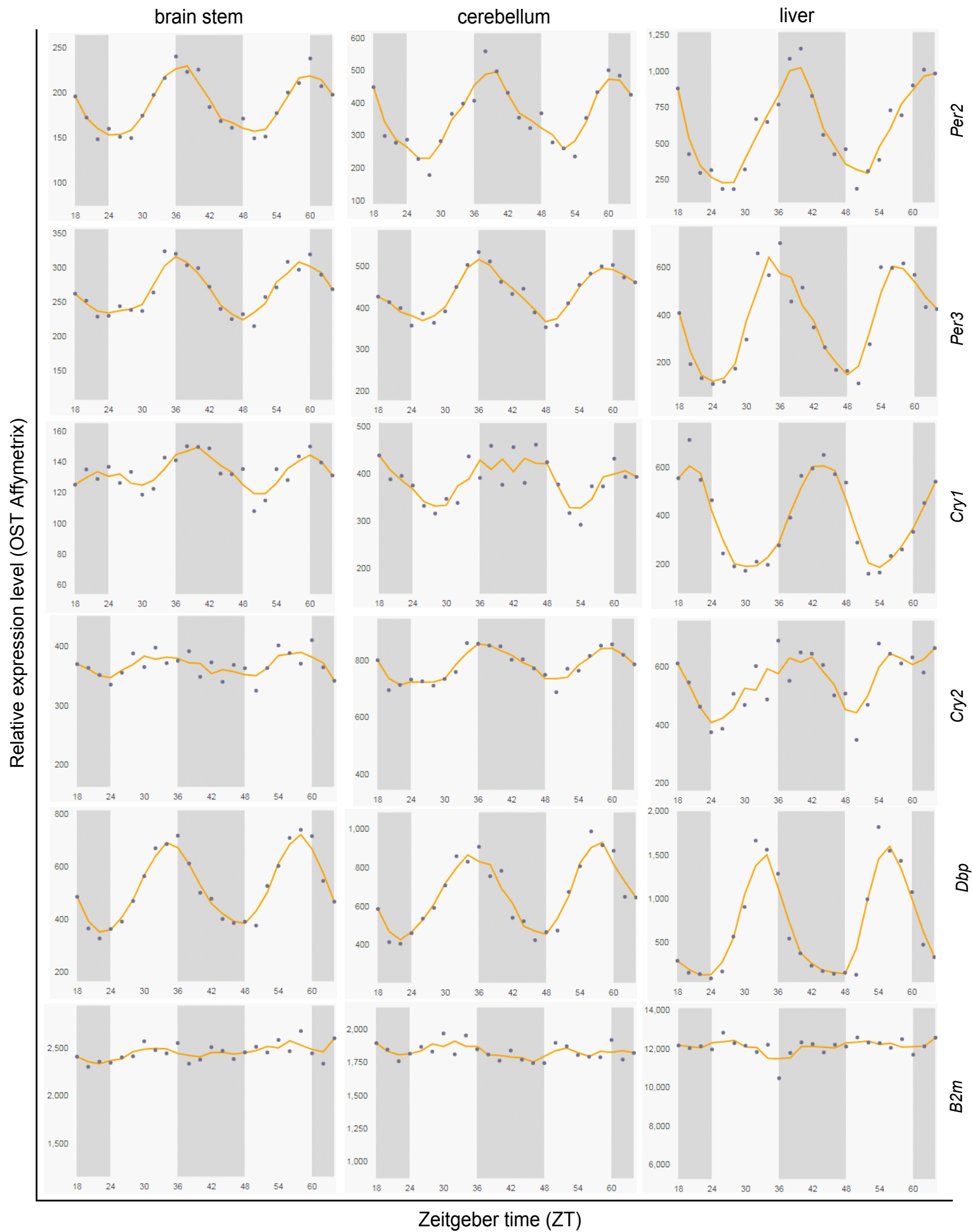

Supplement: S2 Fig — The data are from the publicly available circadian transcriptome database (http://circadb.hogeneschlab.org/mouse). All presented mRNAs show significant circadian oscillations in all three tissues (JTK p<0.05) except Cry1 (non-significant cycling in the brain stem /JTK p = 0.058/) and B2m (no cycling in any tissue /JTK p = 1/). Note that the presented default output graphs from the circadb database show one full 24 h period from ZT24 though ZT48. Therefore, ZT1 or ZT12 in Fig 1 corresponds to ZT25 or 36 in S1 and S2 Figs, respectively. (PDF) [file pone.0249981.s007.pdf]

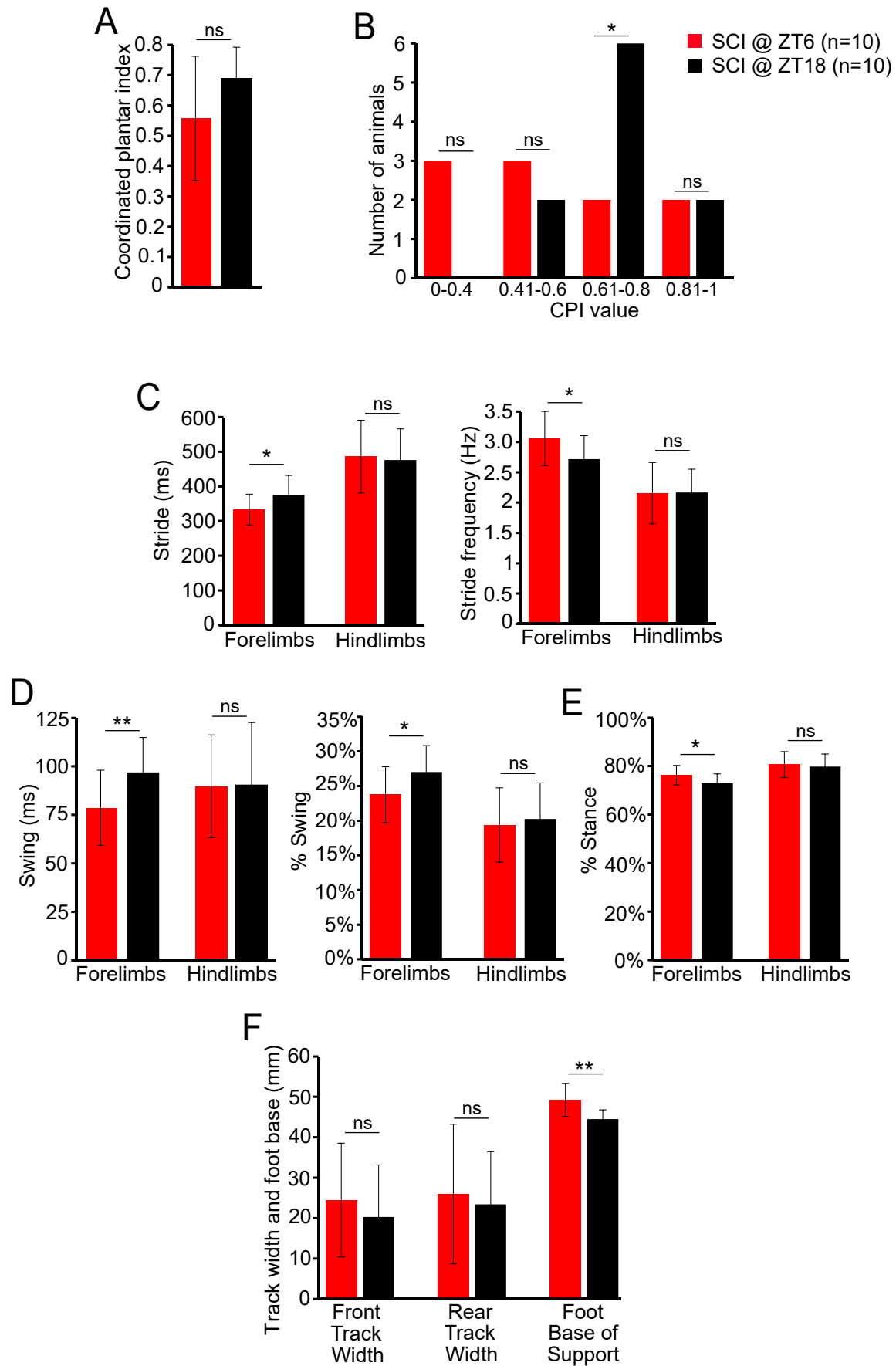

Supplement: S3 Fig — After completion of BMS and ladder walking at week 6 after SCI, gait analysis was performed using the Treadscan system. A-B, Although the average coordinated plantar index (CPI, a ratio between numbers of plantar step cycles with correct sequence of limb placement to all step cycles) was not different between the groups (A), significantly more ZT18 animals achieved high level of coordination (B, CPI>0.6). C-F, Stride analysis revealed no significant differences in hindlimb function with consistent effects on forelimbs suggesting reduced compensatory usage in the ZT18 group. Those include longer and less frequent strides (C), as well as longer swings (D) and shorter stance (E). While the BMS-correlated rear track width was unaffected (Beare et al. 2009, PMID: 19886808), forelimb-hindlimb foot base of support was shorter in the ZT18 group (F). The latter parameter was the only direct indication of potentially improved hindlimb function in ZT18 mice (less hindlimb dragging); additional indirect support for moderate improvement in hindlimb function is provided by the aforementioned lower compensatory usage of forelimbs in that group. Data represent means ± SD; Binominal Proportion test, CPI distribution (z = 2.0, p<0.05, *); RM ANOVA with left vs. right side and time-of-injury used as two factors, effect of time-of-injury: swing (F1,36 = 8.9, p<0.01**), % of swing (F1,36 = 6.3, p<0.05*), % of stance (F1,36 = 6.3, p<0.05*), stride (F1,35 = 6.5, p<0.05*), stride frequency (F1,36 = 6.3, p<0.05*), foot base of support (F1,36 = 14, p<0.01**); ns, p>0.05. Overall, gait parameters suggest only minor effects of time-of-injury on locomotor recovery as compared at ZT6 vs. ZT18. (PDF) [file pone.0249981.s008.pdf]
