# Supplementary material for: Limited changes in locomotor recovery and unaffected white matter sparing after spinal cord contusion at different times of day
Source: PLoS One. 2021 Nov 23;16(11):e0249981. doi: 10.1371/journal.pone.0249981 (PMC8610253; doi:10.1371/journal.pone.0249981)
Supplement: Attachment — Submitted filename: ReviewResponse.docx [file pone.0249981.s009.docx]

RE: Resubmission of the manuscript PONE-D-21-09445

REVIEW RESPONSE:

Reviewer #1, Point # 1: In the title the authors should to be careful when stating circadian time; this manuscript only investigates the effects of Zeitgeber time at two timepoints based around the light cycle.

Response: We replaced the term <circadian time> with either <zeitgeber time> or <time of day>. Also, additional injury timepoints were added for the revision (study 2 examines ZT6 vs. ZT18).

Reviewer #1, Point # 2: There is often a lag before the circadian rhythm responds to changes in the expression of clock regulatory genes, it would be of interest to perform a SCI at other times points. However, the reviewer appreciates that this may be out of the scope of the current manuscript.

Response: We fully agree that comprehensive analysis of time of injury effects should include more timepoints. Therefore, additional injury timepoints were added for the revision (the newly added study 2 examines ZT6 vs. ZT18, Revised Figs. 3 and 4).

Reviewer #1, Point # 3: In Supp. 1 and 2 the Zeitgeber time starts at 18, it would be more relevant to see the differences at 0 to 12 as these are the times useTd in the study.

Response: the graphs in Figs. S1 and S2 were generated as a default output of the circadb database. Each graph covers one full period (ZT24-ZT48). Hence, ZT0 or ZT12 in our data (Fig. 1) corresponds to ZT24 or ZT36, respectively. To avoid confusion, we modified the legend for Fig S1 and S2 to explain that shift.

Reviewer #1, Point # 4: It would be of interest to determine whether the injury resets the clock and induces changes in the expression of clock regulatory genes at the chronic time point.

Response: We agree that this would be an interesting question to answer. However, we feel that this resource-intensive experiment is out of scope of our current paper that is addressing the effects of time of injury on post-injury recovery rather than consequences of the injury for the rhythmicity (to do this correctly with injured animals one would have to study at least 2 periods with 4 probing times/period- 2x4= 8 timepoints per arm). In the context of our data, the most interesting time window for such an experiment would be during acute/subacute phases of recovery when white matter is still being lost and a clock reset could nullify any potential effects of time of injury on lesion size. At least with thoracic level injuries in humans or rats, rhythmicity of various physiological rhythms appears normal at chronic timepoints (PMIDs: 22474242, 21231876, 30627655). Thus, limited chronic outcome could be expected with T9 contusion in mice.

Reviewer #1, Point # 5: In the authors previous publication using Bmal1-/- mice they demonstrate an effect on the integrity of the BSCB and inflammatory response acutely after SCI. It may be interesting to also examine some of these histological markers in this chronic SCI tissue.

Response: We agree that this would be an interesting question to answer. Unfortunately, we did not have any tissue left from our original study (study 1 with SCI at ZT0 and ZT12). However, we generated extra tissue in the new study that probed effects of SCI at ZT6 and ZT18. As in study 2, effects of injury time, while still limited, were greater than those in study 1 (ZT18 group showed small but significant improvement in BMS and better ladder test performance as compared to ZT6), we focused our BSCB/neuroinflammation analyses on that experiment. We have chosen day 7 post injury because at that timepoint BSCB leak is still present, neuroinflammation is well developed, and, Bmal1 deficiency strongly reduced both those processes (Slomnicki et al. Scientific Reports 2020). However, we did not observe any differences between SCI at ZT6 vs. ZT18. Those new data are shown in the revised Fig. 4.

Reviewer #1, Point # 6: In the methods please state at what time of day behavioural assessments were performed.

Response: Modified as requested (all testing was done between 9:00-11:30).

Reviewer #1, Point # 7: Supp.2 the Dbp plot needs to be re-aligned.

Response: Modified as requested.

Reviewer #2, Major concern #1: Clock genes and their downstream pathways usually present a lag in the response to environmental stimuli (Zeitgebers such as light) as also shown by the clock gene expression in Suppl Fig 1 and 2. Indeed, while no difference was found between ZT0 and ZT12, increasing the number of injury time points, i.e. increased temporal resolution, is required to claim that time of day does not play a role, as the authors themselves point out in the discussion.

Response: We conducted another study that compared effects of SCI at ZT6 vs. ZT18. That experiment resulted in similar conclusions as the original experiment with SCI at ZT0 and ZT12. The new data are presented in the revised manuscript as Fig. 3 and Fig. 4.

Reviewer #2, Major concern #2: Since in their previous publication the authors found an effect on the integrity of the BBB and acute inflammatory response in mice with Bmal1 deletion, it would be relevant to examine these aspects in the present study. In fact, here there is no mention of the different cell types present in the intact and injured spinal tissue, what is the clock in the different cell types? And importantly, what happens to them before and after injuries performed at different ZTs?

Response: We agree that defining injury time effects on acute/subacute dysfunction of BBB/BSCB and the neuroinflammatory response would be an interesting question to answer. Therefore, we generated extra tissue in the new study that probed effects of SCI at ZT6 and ZT18. As in that study (study #2), effects of injury time of day on recovery, while still limited, were greater than those in study #1 (ZT18 group showed small but significant improvement in BMS and terminal better ladder test performance as compared to ZT6), we focused our BSCB/neuroinflammation marker analyses on that experiment. We have chosen day 7 post injury 7 because at that timepoint BSCB leak is still present, neuroinflammation is well developed, and, Bmal1 deficiency strongly attenuates those processes (Slomnicki et al. Scientific Reports 2020). However, we did not observe any differences between ZT6 vs. ZT18. Those new data are shown in the revised Fig. 4.

While we agree that defining cell type specific regulation of the clock pathway in the intact and injured spinal cord would be of great interest, performing such a resource and time intensive analysis is beyond the scope of the current manuscript.

Reviewer #2, Major concern #3. Although no difference in functional recovery was found, circadian changes may be subtle and the analysis performed by authors not powerful enough to fully appreciate them, it may be relevant to analyse anatomical changes such as plasticity, regeneration and sprouting of different tracts/neurons affected by injury.

Response: We agree that those additional analyses could provide more insight into subtle time of injury effects on the pathogenesis of SCI. However, such additional experiments would require significant resources and relatively long time to complete. Therefore, their addition would greatly delay timely publication of the current data. To acknowledge a possibility that such additional effects may exist, we added the following statement to the final conclusions paragraph of the discussion:

<However, slightly improved terminal performance in ladder walking after active period SCI suggests subtle, but significant, effects that may involve structural and/or functional post-injury plasticity of spinal cord circuitries.>

Reviewer #2, minor concern #1: The title states “circadian times”, however the injuries are performed on mice kept in a normal LD cycle which makes it impossible to determine circadian time but only zeitgeber time or time of the day

Response: We replaced the term <circadian time> with <zeitgeber time> or <time of day>.

Reviewer #2, minor concern #2: In Suppl Fig 1 and 2 the graphs show a time starting at ZT18, it would be ideal to have them all starting at ZT0, especially considering that the injuries have been performed at ZT0 and ZT12

Response: The graphs in Figs. S1 and S2 were generated as a default output of the circadb database. Each graph covers one full period (ZT24-ZT48). Hence, ZT0 or ZT12 in our data (Fig. 1) corresponds to ZT24 or ZT36, respectively. To avoid confusion, we modified the legend for Fig S1 and S2 to explain that shift.

Reviewer #2, minor concern #3: The time at which the lights are on is usually termed ZT0 and not ZT1. It is not clear why the author performed injury at ZT0 (Fig 2) but show qPCR analysis at ZT1 (Fig 1).

Response: We define ZT0 as the time of lights on (see the Abstract). The difference between the RNA analysis and the SCI study 1 was due to adjustments for staff availability. The rationale of the RNA analysis was to confirm oscillating activity of the clock pathway. That goal was met with the executed experimental design. Also, densely probed mRNA data from the circadb database (Figs. S1, S2), suggest that one hour difference between the experiments would unlikely change the overall conclusions as to the likely status of the clock pathway activity in the ZT0 SCI group.
