# Supplementary material for: Limited changes in locomotor recovery and unaffected white matter sparing after spinal cord contusion at different times of day
Source: PLoS One. 2021 Nov 23;16(11):e0249981. doi: 10.1371/journal.pone.0249981 (PMC8610253; doi:10.1371/journal.pone.0249981)
Supplement: Attachment — Submitted filename: Review_Response.docx [file pone.0249981.s010.docx]

**RE: Resubmission of the manuscript PONE-D-21-09445R1**

**REVIEW RESPONSE:**

REVIEWER #1: POINT 1: The new results for the normalised horizontal ladder in both study 1 and 2 appear confusing. The mice injured at ZT12 or ZT18 do not recover significantly more but rather that the mice injured at ZT0 or ZT6 get significantly worse at 6 weeks, do the authors have any suggestions in why these groups would deteriorate at week 6?
I can appreciate why the authors tried to normalized the horizontal ladder data due to high variability. However, this is an unusual and confusing way to analyse this data and I believe sufficient number of animals were used to observe real behavioural differences between the groups. I would recommend to show the unnormalized analysis using number of errors, as was provided in the first submission.

RESPONSE: We included the “raw” error number data for both study 1 (Fig. 2B) and study 2 (Fig. 3B) as requested. The normalized data are shown as well (Fig. 2B’ and 3B’). Results description and figure legends were modified accordingly (highlighted in the track change version of the manuscript). Edits were introduced to those sections to clearly define what the normalized error score is (in the revised manuscript we referrer to that parameter as a change in error number from the previous testing). The discussion section contains a paragraph that presents the case for post-injury spasticity as a potential driver for week 6 worsening in ladder test performance of animals with daytime injuries (lines 342-353).

REVIEWER #1, POINT 2: On Line 264 the authors state “Both BMS (A) and normalized error score in the horizontal ladder walking test (B) revealed minor yet significant improvement in locomotor recovery between ZT18 and ZT6.” But the authors go on to say that “For BMS, no significant group differences were observed with post hoc testing at any time point” Please clarify and correct the statement and results of the BMS. The data appears to show an improvement with time, due to spontaneous recovery but no differences between the groups.

RESPONSE: we apologize for those confusing statements. No significant effects of time of day on BMS were observed in study 1 or study 2. The sentences in question were all modified accordingly.

REVIEWER #1, POINT 3: Investigating the BSCB and neuroinflammation markers is a welcomed addition to the paper. It may also be interesting to assess fluorescence intensity of these markers at the lesion site. For instance, in the image in Fig.4 CD45 staining appears to be more intense at ZT18.

RESPONSE: we agree that quantifying the BSCB/neuroinflammation markers in a region- specific manner could be potentially interesting. However, given the overall negative nature of our findings and preferable use of signal area quantifications rather than signal intensity we do not expect that such analyses would affect our conclusions in a major way. To avoid confounding effects of staining to staining variability that can affect intensity we prefer signal area analyses. Thus, the apparent difference in CD45 signal intensity is unlikely to change results of the area analyses as shown in Fig. 4B.

REVIEWER 1, POINT 4: The figure legend and methods for Fig.4 states that coronal sections were used for staining and image analysis yet the images in Fig.4 appear to be longitudinal images, please clarify this.

RESPONSE: We use the term “coronal” to describe the sections that are cut in the coronal plane of the human body that in the spinal cord would include its long axis. We recognize that this may be confusing as the term “longitudinal” is usually used throughout the literature to describe such sections. To avoid such confusion, we replaced the term coronal with longitudinal.
